# A mathematical description of fossilization

**DOI:** 10.1098/rsos.231827

**Published:** 2024-07-17

**Authors:** Corentin C. Loron

**Affiliations:** ^1^ School of Physics and Astronomy, University of Edinburgh, Edinburgh, UK

**Keywords:** fossils, taphonomy, decay, diagenesis, palaeontology, Price equation

## Abstract

Fossils constitute an inestimable archive of past life on the Earth. However, the stochastic processes driving decay and fossilization and overwhelmingly distorting this archive, are challenging to interpret. Consequently, concepts of exceptional or poor preservation are often subjective or arbitrarily defined. Here, we offer an alternative way to think about fossilization. We propose a mathematical description of decay and fossilization relying on the change in the relative frequency and characteristics of biogenic objects (e.g. atoms, functional groups, molecules, body parts and organisms) within an organism–fossil system. This description partitions taphonomic changes into three categories: gain, loss and alteration of state. Although the changes undergone by organisms through decay, preservation and alteration vary a lot for different organisms under different conditions, we provide a unified formalism which can be applied directly in the comparison of different assemblages, experiments and fossils. Our expression is closely related to George R. Price’s famous equation for the change in evolutionary traits and can be adapted to the study of palaeontological systems and many others.

## 1. Introduction

Taphonomy, a subdiscipline of palaeontology, archaeology and forensic science, is the study of the degradation of living matter in natural environments and, ultimately, its translation from the biosphere into the geosphere [1]. To identify and characterize extinct life forms from the rock record, taphonomists investigate the post-mortem, pre- and post-depositional processes, whereby organisms are modified by their physico-chemical environment (e.g. microbial activity, diagenetic alteration and metamorphism [2]).

Evidently, the original composition of the organism also plays a fundamental role in its taphonomic trajectory in a given setting. The composition of the biological organic material buried in sediment depends on the stability, resistance and solubility of its constituents and upon the extent of biological and chemical attack [3]. The remaining fraction, if not rapidly incorporated into the sediment, will undergo various condensation reactions forming new polymeric material, like the formation of melanoidin-like compounds via the Maillard reaction [4–6], although the global contribution of such compounds to the final sedimentary organic mix has been questioned [7]. Survival of labile material can be observed, notably by association with more resistant molecules acting as ‘shelter’ (e.g. lipids [8]), but the preservation of pristine or lightly altered organic precursors in a fossil remains an exception. Therefore, the survival of biomolecules could not be solely explained by strong preservation potentials and selective preservation pathways [2,3,9].

Considering all this, exceptional morphological preservation cannot be automatically synonymous with exceptional molecular preservation. For example, the exceptional state of preservation observed for many Ediacaran fossils is considered the result of microbially mediated mineral formation resulting in the detailed cast of the organism (the ‘death mask’ model [10,11]) but does not directly preserve the original organic material (with notable exceptions [12]). As a result, defining ‘exceptional’ preservation within Konservat–Lagertätten may be subjective, and depends on the chosen point of view.

However, whatever the biological and environmental settings, fossilization (and to a wider extent, quality of preservation) can be abstracted as a change occurring between two systems: an organism and its decayed—or its fossil—counterpart. Insights from taphonomy and decay experiments [2,3,13–17] and molecular investigation of fossil (e.g. [6–8,18–22]) show that post-mortem processes acting on any organisms or biological remains can be broken down into three broad categories of change: (i) gain of features (deposition of new material, including molecular and mineral), (ii) loss of features (e.g. disappearance of body parts or molecular components), and (iii) modification of the state of the features (decay and alteration). From this perspective, fossilization can have a general mathematical definition.

Building on this thought, we derive here a general mathematical expression, showing similarity with the Price equation in evolutionary biology, that allows the description of the taphonomic process in terms of changes in frequency and characteristics of a set of objects. By describing how the value of a chosen character can change between an organism and its fossil, this expression offers a quantifiable partition of post-mortem dynamics.

## 2. Mathematical framework

### 2.1. Definitions

Consider two multi-component entities *Q* and *Q*′. Let *q_i_* be the relative frequency of the *i*th object in *Q* and *q*′_*i*_the relative frequency (hereafter designated as frequency) of the *i*th object in *Q′*. For our narrative, *Q* and *Q′* may represent the source organism and its fossil, respectively. The objects are any unit of interest within these entities, for example, atoms, functional groups, molecules, tissues or body parts. Alternatively, *Q* and *Q*′ can represent a living population and its fossil counterpart.

We take


(2.1) $$q\prime_{i}=c_{i}q_{i},$$

with *c* representing the factor of the *i*th object by which *q_i_* has been modified in *q′_i_* such that $c_{i}=\frac{q\prime_{i}}{q_{i}}$.


(2.2) $$\overset{-}{c}=\sum q_{i}c_{i}=\sum q_{i}\frac{q\prime_{i}}{q_{i}}=\sum q\prime_{i}=1.$$

Let *s*_*i*_ be the state of an object in *Q* and *s′*_*i*_ the state of an object in *Q′*. A state here represents the value of any characteristic of interest, for example, a size, a weight, a surface or any other variable obtained by qualitative or quantitative measurement (e.g. length of the carbon chain, percentage of tissue decay and tissue hardness). We have the average (here and after understood as the expected value) value of *s*,


(2.3) $$\overset{-}{s}=\sum q_{i}s_{i}.$$

Similarly,


(2.4) $$\overset{-}{s}\prime =\sum q\prime_{i}s\prime_{i}$$

represents the average state value of the objects constituting an organism as it becomes a fossil. The change in the state value of *s_i_* in *s′_i_,* $\Delta s_{i}$, is


(2.5) $$\Delta s_{i}=s\prime_{i}-s_{i}.$$

Equally,


$$s\prime_{i}=s_{i}+\Delta s_{i}.$$

For the whole system (e.g. organism fossil), the average change in state value for a chosen metrics, $\Delta \overset{-}{s},$ can be written as


(2.6) $$\Delta \overset{-}{s}=\overset{-}{s^{\prime }}-\overset{-}{s}.$$

### 2.2. Derivation

With the definitions above, we can derive $\Delta \overset{-}{s}$ between *Q* and *Q′* as


(2.7) $$\Delta \overset{-}{s}=\overset{-}{s^{\prime }}-\overset{-}{s},$$

(2.8) $$\;\sum q_{i}^{\prime }s_{i}^{\prime }-\sum q_{i}s_{i},$$

(2.9) $$\;\sum q_{i}^{\prime }\left(s_{i}+\Delta s_{i}\right)-\sum q_{i}s_{i},$$

(2.10) $$\;\sum c_{i}q_{i}s_{i}+\sum c_{i}q_{i}\Delta s_{i}-\sum q_{i}s_{i}.$$

For convenience, we switch the order of the terms and have


(2.11) $$\Delta \overset{-}{s}=\;\sum c_{i}q_{i}s_{i}-\sum q_{i}s_{i}+\sum c_{i}q_{i}\Delta s_{i}.$$

Multiplying the second term by $\overset{-}{c}$ , we can rewrite $∆\overset{-}{s}$ as


(2.12) $$\Delta \overset{-}{s}=\;\sum c_{i}q_{i}s_{i}-\sum q_{i}c_{i}\sum q_{i}s_{i}+\sum c_{i}q_{i}\Delta s_{i}.$$

Rewriting $\Delta \overset{-}{s}$ as a sum of expectations we obtain


(2.13) $$\Delta \overset{-}{s}=\;Ec_{i}s_{i}-Ec_{i}Es_{i}+Ec_{i}\Delta s_{i}.$$

By considering $Ec_{i}s_{i}-Ec_{i}Es_{i}=Cov\left[c_{i},s_{i}\right]$, we arrive at an expression in the same form as the famous Price equation for evolutionary changes [23–26],


(2.14) $$\Delta \overset{-}{s}=\;Cov\left[c_{i},s_{i}\right]+Ec_{i}\Delta s_{i}.$$

In this way, the taphonomic changes can be statistically described as the change in the average value of the state of an agent. Here, equation (2.14) represents the changes in the frequency of objects without alteration (first term) and the alteration of the states of existing objects (second term). The covariance term can also be expressed as the product of a variance and a regression coefficient [23,27]. If this coefficient is positive (or, equivalently, if the covariance term is positive), the frequency of a specific state value is expected to rise in the second entity. If this term is null, then no changes are observed in the frequency of objects between the entities.

Considering the presence of new objects in *Q′* absent in *Q* (e.g. incorporation of external objects or formation of new objects) requires a small redefinition of our system. Let us now take Q*, such that Q* plus *Q′* represents the second entity. All objects in *Q′* were originally in *Q*, whereas the objects in Q* are the objects that are not found originally in *Q*. Let *p* be the probability that a given object belongs to Q* rather than *Q′*. We have now


(2.15) $$\overset{-}{s^{\prime }}=p\sum q_{i}^{*}s_{i}^{*}+\left(1-p\right)\sum q_{i}^{\prime }s_{i}^{\prime },$$

with $\sum q_{i}^{*}s_{i}^{*}$ the expected value of s in Q*

Returning to $\Delta \overset{-}{s}$, we have


(2.16) $$\Delta \overset{-}{s}=\overset{-}{s^{\prime }}-\overset{-}{s},$$

(2.17) $$p\sum q_{i}^{*}s_{i}^{*}+\left(1-p\right)\sum q_{i}^{\prime }s_{i}^{\prime }-\sum q_{i}s_{i},$$

(2.18) $$p\sum q_{i}^{*}s_{i}^{*}+\left(1-p\right)\sum q_{i}^{\prime }s_{i}^{\prime }-\left[p+\left(1-p\right)\right]\sum q_{i}s_{i},$$

(2.19) $$p\sum q_{i}^{*}s_{i}^{*}+\left(1-p\right)\sum q_{i}^{\prime }s_{i}^{\prime }-p\sum q_{i}s_{i}+\left(1-p\right)\sum q_{i}s_{i},$$

(2.20) $$p\left(\sum q_{i}^{*}s_{i}^{*}-\sum q_{i}s_{i}\right)+\left(1-p\right)\left(\sum q_{i}^{\prime }s_{i}^{\prime }-\sum q_{i}s_{i}\right).$$

Using the definitions above, we have


(2.21) $$\Delta \overset{-}{s}=p\left(\Delta {\overset{-}{s}}^{*}\right)+\left(1-p\right)\left(Cov\left[c_{i},s_{i}\right]+Ec_{i}\Delta s_{i}\right).$$

We rearrange and develop the terms to obtain a three-term equation,


(2.22) $$\Delta \overset{-}{s}=\left(1-p\right)Cov\left[c_{i},s_{i}\right]+\left(1-p\right)Ec_{i}\Delta s_{i}+p\left(\Delta {\overset{-}{s}}^{*}\right).$$

**Figure 1.**
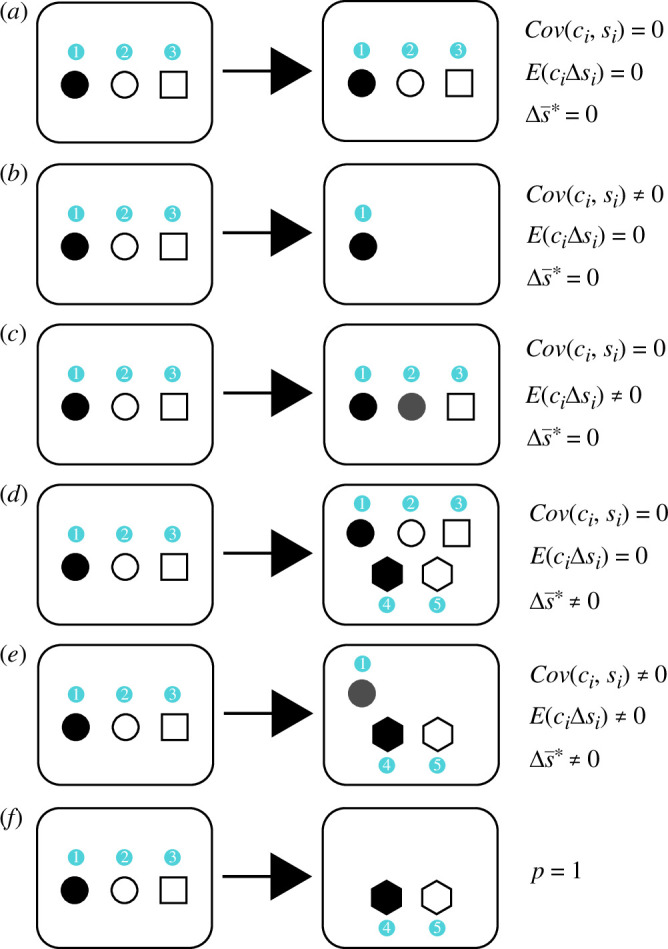
Partitions of taphonomical effects between an organism and its fossil counterpart. Here, shapes represent different numbered objects and shading (white, black and grey) represents different state values. (*a*)–(*c*) illustrate situations solely accounting for changes in frequencies and state values (similar to the general Price equation). The probability *p* of having additional objects is 0 and the term $\Delta {\overset{-}{s}}^{*}$ vanishes. (*d*)–(*f*) illustrate the extended Price equation presented here, accounting for the change owing to the properties of the objects in the original organism plus the products of post-mortem condensation reactions and incorporation of external material. Probability *p* is non-zero if new objects are present and equal to 1 when the fossil no longer contains any of the original material (*f*).

The effects of different partitions on the terms in equation (2.22) (and equation (2.14) above) are illustrated in figure 1. Altogether, the three terms account for all possible taphonomic modifications (gain, loss and alteration of features), including the total replacement of the original material. As displayed in figure 1*a*, for an ideal preservation (*Q′* is the exact image of *Q*), all terms would vanish. This is owing to the absence of alterations and additions and the preservation of each object in similar frequencies in both entities. Such a situation is not physically possible if we consider an organism as a whole. Nevertheless, it is possible that a component of interest may remain pristine or with only a few alterations (figure 1*c*) or that the only change may be additive (for example, the precipitation of mineral components; figure 1*d*).

On the other hand, we can consider several different ways in which a fossil might be said to be poorly preserved. Most simply, poor preservation could be characterized by a low factor of retention of original objects (*c* tending to 0; figure 1*b*). We can also describe a situation whereby all remaining objects in *Q′* (regardless of their preservation potential) have changed from their original value (e.g. figure 1*e*). In this state of total alteration, and for all *i*,


(2.23) $$\Delta s_{i}=s\prime_{i}-s_{i}\neq 0\;\forall i.$$

Finally, if there are no common objects between the two entities, i.e. if *p* = 1 (figure 1*f*), then the first two terms in equation (2.22) vanish and


(2.24) $$\overset{-}{s^{\prime }}=\sum q_{i}^{*}s_{i}^{*}$$

is a lower bound for the retention of information in a fossil about its source organism (although, in this case, we do not really have a fossil at all!).

## 3. Examples

We now provide examples of using equations (2.14) and (2.22) with empirical data.

### 3.1. General examples

**Figure 2.**
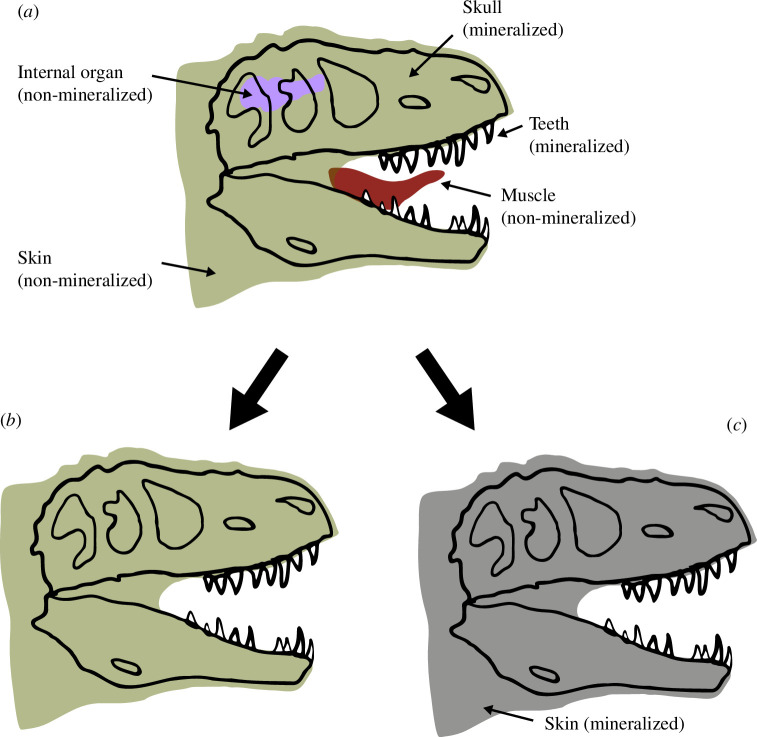
Illustration of the Price equation formalism adapted to taphonomical systems. Here, the *T. rex* head in (*a*) is composed of three soft-tissue, non-mineralized parts (skin, internal organ and muscle) and hard-tissue, mineralized skull and teeth. A hardness value *s* of 1 is assigned to non-mineralized parts and 2 otherwise. In the first fossil (*b*), the organ and muscle have not been preserved, reducing the frequency of soft tissues compared with the living organism. Conversely, the skull and teeth have been preserved, so hard tissue frequency has increased. (*b*) Illustrates the covariance term of equations (2.14) and (2.22) and accounts for selective preservation. In fossil (*c*), the frequency of original soft and hard tissues is the same than in (*b*) but, the preserved soft tissue (skin) has been altered and is now also mineralized (its hardness value has passed from 1 to 2; but it is still originally a soft tissue). This example represents the expectation term of the equations that account for alteration.

Let us take an organism composed of five body parts: a skull, teeth, the skin, a muscle (tongue) and an internal organ; the brain of an imaginary *Tyrannosaurus rex* (figure 2*a*). We can divide these parts into two groups: hard-tissue (mineralized teeth and skull), for which we measured a hardness value of 2, and soft-tissue (non-mineralized skin, muscle and brain), with a lower value of 1. Note that the choice of hardness values 1 and 2 is here made for simplicity; the value from any metrics may be chosen instead. Their respective frequencies are:

Non-mineralized (*q_nm_*): 3/5

Mineralized (*q_m_*): 2/5

We start with


$$\overset{-}{s}=\sum q_{i}s_{i}=\;q_{nm}s_{nm}+q_{m}s_{m}=\frac{3}{5}1+\frac{2}{5}2=\frac{7}{5}.$$

In our first scenario (figure 2*b*), we preserve the mineralized parts and partially preserve the non-mineralized parts. The updated frequencies are:

Non-mineralized (*q′_nm_*): 1/3

Mineralized (*q′_m_*): 2/3

The *c* values between the two sets of frequencies are


$$c_{nm}=\frac{q\prime_{nm}}{q_{nm}}=\frac{1/3}{3/5}=\frac{5}{9},$$

$$c_{m}=\frac{q\prime_{m}}{q_{m}}=\frac{2/3}{2/5}=\frac{5}{3}.$$

The updated $\overset{-}{s\prime }$ is


$$\overset{-}{s\prime }=\sum q\prime_{i}s\prime_{i}=q\prime_{nm}s\prime_{nm}+q\prime_{m}s\prime_{m}=\frac{1}{3}1+\frac{2}{3}2=\frac{5}{3}.$$

Here, the state values of the body parts of our fossil are unchanged compared with the living organism ($Ec_{i}\Delta s_{i}=0$). There is no new material so we may use equation (2.14) directly. We have


$$\Delta \overset{-}{s}=\;Cov\left[c_{i},s_{i}\right]+Ec_{i}\Delta s_{i},$$

$$\left[q_{nm}\left(s_{nm}-\overset{-}{s}\right)\left(c_{nm}-\overset{-}{c}\right)+q_{m}\left(s_{m}-\overset{-}{s}\right)\left(c_{m}-\overset{-}{c}\right)\right]+0,$$

$$\frac{3}{5}\left(1-\frac{7}{5}\right)\left(\frac{5}{9}-1\right)+\frac{2}{5}\left(2-\frac{7}{5}\right)\left(\frac{5}{3}-1\right)=0.266,$$

which is equivalent to $\Delta \overset{-}{s}=\overset{-}{s^{\prime }}-\overset{-}{s}=\frac{5}{3}-\frac{7}{5}=0.266$, demonstrating the tautological nature of the Price equation [26]. This first scenario (figure 2*a*) illustrates how the first component of equations (2.14) and (2.22) relates to the part of the change solely owing to the variation in frequencies of the objects. This variation is factored by *c*, which effectively acts as a fitness function: the resistant components (here the mineralized skull and teeth) have better *c* factor than more labile non-mineralized skin, muscle and organ (*c*_m_ = 5/3 ≈ 1.67 and *c*_nm_ = 5/9 ≈ 0.56) and are better represented in the fossil. Therefore, $Cov\left[c_{i},s_{i}\right]$ quantifies the preservation and loss of objects during the taphonomic process solely owing to the original intrinsic characteristic of the type of objects and their decay/degradation resistance. Because this covariance term includes a direct expression (*c*) of the relation between the frequencies of the objects in the two entities, i.e. a growth factor, it can be considered as a selection covariance [27,28]. Thus, this first term is a direct expression of selective preservation.

For our second scenario (figure 2*c*), we now consider some changes in the hardness values of our body parts in the fossil (we retain the change of frequencies from the previous scenario). The only preserved soft tissue, the skin, has been altered post-mortem and has been mineralized. Its hardness value has changed from 1 to 2. The updated $\overset{-}{s\prime }$ is now


$$\overset{-}{s\prime }=\frac{1}{3}2+\frac{2}{3}2=2.$$

We now have


$$\Delta \overset{-}{s}=\;Cov\left[c_{i},s_{i}\right]+Ec_{i}\Delta s_{i},$$

$$\;0.266+\sum q_{i}c_{i}\left(s_{i}^{\prime }-s_{i}\right)=\;0.266+\sum q\prime_{i}\left(s_{i}^{\prime }-s_{i}\right),$$

$$\;0.266+\left[\frac{1}{3}\left(2-1\right)+\frac{2}{3}\left(2-2\right)\right]=0.266+\frac{1}{3}=0.596.$$

Here, the second term, $Ec_{i}\Delta s_{i}$, describes the part of the change that is owing to variation in state values (the skin hardness of 1 becoming 2 after getting mineralized). In our *T. rex* scenario, we can see that this term scores approximately 0.33 ($\frac{1}{3}$), whereas our first term retains its score of 0.266 (no change in frequency). For simple visualization, we could imagine converting these two positive values as percentages of the total score, 0.596, which means that alteration accounts for 55% of the observed change in hardness between the organism and the fossil. On the other hand, the selective preservation of the mineralized hard tissues accounts for 45% of it. In nature, such changes may have many causes, among which are microbial, chemical and physical activities. The second term is an expression of the modification of our object by all these processes. It is the alteration term.

**Figure 3.**
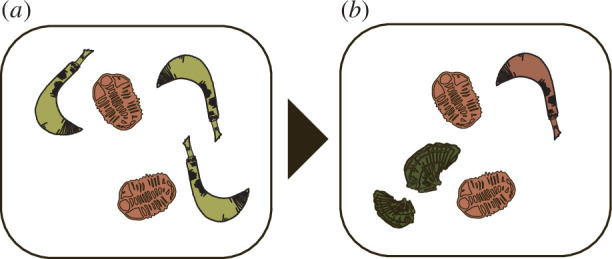
Illustration of the present formalism. The assemblage (*a*) is composed of three soft-bodied and two hard-bodied organisms (of hardness value 1 (yellow) and 2 (orange), respectively) and degrades into assemblage (*b*), composed of the same two hard-bodied fossil (value 2), one soft-bodied fossil (altered hardness value of 2) and two new fossils, transported in the assemblage from elsewhere (hardness value of 3 (green)). The state value of these new objects is accounted for in the last term of equation (2.22), the incorporation term.

To illustrate the third term of equation (2.22), we take another simple example. Let us now consider whole organisms in a depositional environment (figure 3*a*). Our living community is composed of five organisms: three soft-bodied *Ottoia* (a priapulid worm [29]) and two hard-bodied trilobites. Their respective frequencies are:

*Ottoia* (*q_o_*): 3/5

Trilobite (*q_t_*): 2/5

Just as with the previous examples, we can characterize the change in our system as being owing to the selective preservation of hard tissues and alteration (e.g. soft tissues becoming mineralized). We kept the same frequency and values as on the first examples ($Cov\left[c_{i},s_{i}\right]=0.266$ and $Ec_{i}\Delta s_{i}=\;\frac{1}{3}$); however, this time, our fossil assemblage (figure 3*b*) also contains two shell fragments transported from elsewhere and with *s* = 3. The group of new objects is composed of a single type (the shells) with $q_{n}^{*}=\;1.$ In our fossil assemblage, the probability of belonging to this new group is $p=\;\frac{2}{5}$ . The updated $\overset{-}{s^{\prime }}$ is


$$\overset{-}{s^{\prime }}=p\sum q_{n}^{*}s_{n}^{*}+\left(1-p\right)\sum q_{o,t}^{\prime }s\prime_{o,t},$$

$$\frac{2}{5}\left(1*3\right)+\frac{3}{5}\left(\frac{1}{3}2+\frac{2}{3}2\right)=2.4.$$

As per equation (2.22), we now have


$$\Delta \overset{-}{s}=\left(1-p\right)Cov\left[c_{i},s_{i}\right]+\left(1-p\right)Ec_{i}\Delta s_{i}+p\left(\Delta {\overset{-}{s}}^{*}\right),$$

$$\frac{3}{5}0.266+\frac{3}{5}*\frac{1}{3}+\frac{2}{5}\left(3-\frac{7}{5}\right)=0.999.$$

The third term, $\Delta {\overset{-}{s}}^{*}$, accounts here for the change owing to the appearance or migration of new objects into the system that were not present in the original entity. In our example, it represents the change induced by the transportation of external organisms to our depositional environment. In other systems, for example when investigating post-mortem molecular dynamics (where molecules or functional groups are taken as objects), it would account for the part of the change resulting from *in situ* polymerization and condensation of new material, the precipitation of minerals and migration of material from the sediment and porewaters. It is the incorporation term.

With equation (2.22), we are now able to express the partition of the taphonomical change of one state. Following our last example above, we have our first, second and last terms respectfully scoring approximately 0.16, 0.2 and 0.64 (weighted by the probability of new material) and a total value of approximately 1. Consequently, the change in hardness value between the extant and fossil assemblage is 16% owing to the selective preservation of hard tissues, 20% owing to the alteration of existing material and 64% owing to the transport of external material.

### 3.2. Specific examples

**Figure 4.**
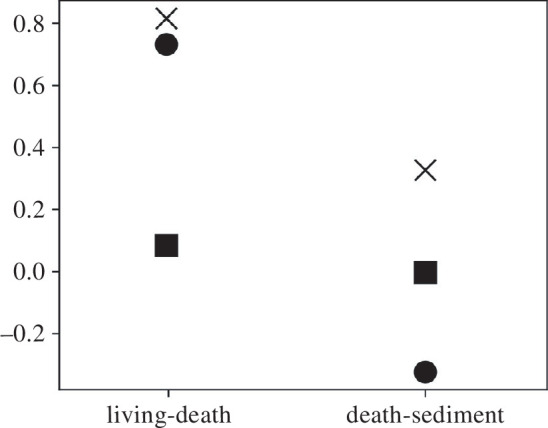
Partition of the post-mortem changes of an epibenthic community from living to death assemblage and from death to sediment assemblage. Crosses indicate the absolute total change, $|\Delta \bar{s}|$; circles indicate the selection term of equation (2.22), **$\left(1-p\right)Cov\left[c_{i},s_{i}\right]$** and squares indicate the incorporation term, $p\left(\Delta {\overset{-}{s}}^{*}\right)$. Original data are from [30] (see the electronic supplementary material).

The previous examples can be extended to describe and partition the effect of taphonomy in various real-time scenarios. For example, Zuschin *et al.* [30] interrogate the post-mortem trajectories of epibenthic communities by comparing the contribution of the organisms in a living assemblage (epifauna) with a death assemblage (benthic islands) and the sediment composition. They observe that the selective preservation of mineralized organisms is the main taphonomic driver to the benthic islands but that fragile/lightly mineralized organisms are the main contribution to the sediment. The sediment fraction also contains organisms that do not contribute to the benthic islands (e.g. vagile crustaceans and sponge spicules) [30]. To study the change in composition from the living assemblage to the death assemblage and from the death assemblage to the sediment, we assigned for each category of objects in [30] (see the electronic supplementary material) a value corresponding to their level of mineralization (1 = soft-bodied; 2 = fragile; 3 = well mineralized). These values are not expected to change between each transition, so the second term ($Ec_{i}\Delta s_{i}$) is null. Applying equation (2.22), we observe that the change is more important between the living and the death assemblage ($|\Delta \bar{s}|$ = 0.816) than between the death and the sediment assemblages ($|\Delta \bar{s}|$ = 0.327), as expected in [30] (figure 4). The fact that well-mineralized organisms are not selectively preserved from death to the fossil assemblage (negative $Cov\left[c_{i},s_{i}\right]$) also supports the conclusion in [30]. In addition, the effect of incorporated objects in the death assemblage (tubes and rocks) and the sediment assemblages (crustaceans, sponges and others) play only a limited role in the whole taphonomic change ($p\left(\Delta {\overset{-}{s}}^{*}\right)$ = 0.083 and −0.003, respectively) compared with the change in object frequencies ($\left(1-p\right)Cov\left[c_{i},s_{i}\right]$ = 0.732 and −0.324, respectively).

**Figure 5.**
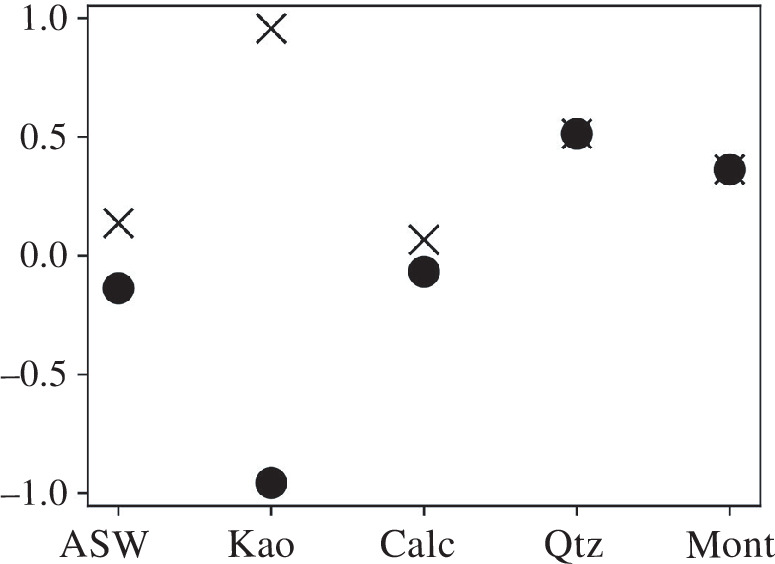
Effects of sediment mineralogy on preservation potential of molecular components of the polychaete, *N. virens*. Crosses indicate the absolute total change, $|\Delta \bar{s}|$ and circles indicate the selection term of equation (2.14), $Cov\left[c_{i},s_{i}\right]$, which is the only contributor to the taphonomic change, $\Delta \overset{-}{s}$, here. ASW: artificial sea water; Kao: kaolinite; Calc: calcite; Qtz: quartz; Mont: montmorillonite. Original data are from [31,32] (see the electronic supplementary material).

Changes in composition during taphonomy can also be tracked within a single organism. Wilson & Butterfield [31] investigate the changes in the molecular composition of a polychaete, *Nereis virens*, buried for four months in various environmental conditions (artificial seawater, kaolinite, calcite, quartz and montmorillonite) to test the effects of sediment mineralogy on preservation potential. Their results show that the fossilization potential of tissues depends on the interplay of the organic composition and the early diagenetic conditions more than on alleged recalcitrance. We assigned a value to each of the studied molecular types based on their alleged recalcitrance [3] (water-soluble proteins = 1; recalcitrant proteins = 2; carbohydrates = 3; chitin = 4; lipids = 5). For this example, we consider these values to be stable during decay ($Ec_{i}\Delta s_{i}=0$). Similarly, there is no incorporation of a new component ($\Delta {\overset{-}{s}}^{*}=0$). Using equation (2.14), we calculate a larger departure from the original composition of *N. virens* (as defined in [32]) in kaolinite ($|\Delta \bar{s}|$ = 0.957), quartz (0.513) and montmorillonite than in calcite (0.067) and artificial seawater (ASW; 0.137). However, the negative covariance shows that molecules with high preservation potentials (lipids and structural carbohydrates) are selected against ASW, kaolinite and calcite ($Cov\left[c_{i},s_{i}\right]$ = −0.137, −0.957, and −0.067, respectively; figure 5), supporting Wilson & Butterfield's [31] results.

**Figure 6.**
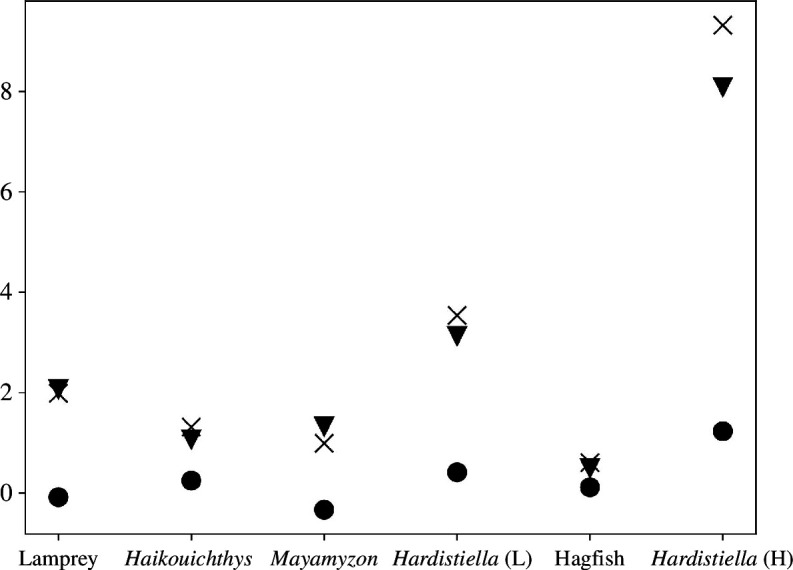
Illustration of the post-mortem plesiomorphic drift (stem-ward slippage) in decayed (lamprey and hagfish) and fossils (*Haikouichthys*, *Mayamyzon* and *Hardistiella*) cyclostomes. *Hardistiella* (L) and *Hardistiella* (H) indicate that the fossil is compared with living lamprey and hagfish, respectively (see fig. 2 in [33]). Crosses show the absolute total change, $|\Delta \bar{s}|$; circles are the selection term of equation (2.14), $Cov\left[c_{i},s_{i}\right]$, and triangles indicate the alteration term, $Ec_{i}\Delta s_{i}$. Original data are from [33] (see the electronic supplementary material).

Distortions of the original composition, or appearance, between an organism and its fossil can lead to an erroneous interpretation of its nature. For example, Sansom *et al.* [33] observed that, after decay, specimens of cyclostomes (lamprey and hagfish) appear morphologically more primitive (plesiomorphic). They also compared various fossils with the decayed specimens to clarify their putative taxonomic affinities. We group the studied characters in [33] by their taxonomic levels and assign each group a value corresponding to their mean synapomorphic ranks (see supplementary file in [33]). The higher the value, the more plesiomorphic the taxonomic rank. Using equation (2.22), we observe that plesiomorphic characters seem to be selected against in decayed lamprey (negative $Cov\left[c_{i},s_{i}\right]$ = −0.086) compared with decayed hagfish ($Cov\left[c_{i},s_{i}\right]$ = 0.111) (figure 6). For both specimens, the selection term also contributes less to the change than the alteration term ($Ec_{i}\Delta s_{i}=$ 2.070 for the lamprey and $Ec_{i}\Delta s_{i}=$ 0.491 for the hagfish). Here, both terms account for the effects of decay on the organism, but the higher values for the alteration term indicate that the observed plesiomorphic appearance is due more to the change in value for the groups of characters being preserved than to simple selective preservation of plesiomorphic characters. When compared with non-decayed lamprey, fossil specimens of *Haikouichthys*, *Mayamyzon* and *Hardistiella* also present higher alteration than selection ($Ec_{i}\Delta s_{i}$ = 1.066, 1.323 and 3.128 against $Cov\left[c_{i},s_{i}\right]$ = 0.245, –0.335 and 0.410, respectively) (figure 6), although selective preservation seemed to have contributed more to their present plesiomorphic appearance (note that only the preserved characters were considered here, see fig. 2 in [33]). Finally, fossil *Hardistiella* presents less difference with lamprey than with hagfish ($|\Delta \bar{s}|$ = 3.538 and 9.323, respectively), as shown by Sansom *et al.* [33] (figure 6).

## 4. Discussion

It is now well understood that selective preservation cannot solely account for the survival of organic material through geological ages [2,9,34]. This implies that taphonomic change is partitioned. In the present work, we mathematically decompose this change into three terms accounting for each aspect of decay and fossilization. The resulting equation (2.22) is an elaboration of the Price equation [23–28] with a third term accounting for the incorporation of new material (e.g. migration from the sediment, neoformation of minerals and transport). Indeed, the original expression of the Price equation is limited to changes occurring between a descendant and their respective ancestors [27].

Kerr & Godfrey-Smith [35] and Fox & Kerr [36] derived an equation resembling equation (2.22) above, which they applied to the expression of total change in ecosystem function. Their expression is built upon the connectedness of the objects between the two studied sites and considers the effect of loss and migration of species. The expression from Kerr Godfrey-Smith [35] and Fox Kerr [36], along with equation (2.22) above, can extend the use made of the Price equation in evolutionary palaeontology [37,38] to the study of extinction events and speciation dynamics where the extra term can account for the appearance of new species. In a similar way, the present expression (equation (2.22)) extends it to the study of fossil assemblages and depositional environments, providing insight into the bias of preservation owing to the organisms themselves (e.g. presence of hard resistant mineral parts or not), the environmental conditions of the site; or the transport of external organisms to the depositional site, as demonstrated above (the first example in §3.2, figure 4). The same object placed to decay in variable settings may provide widely different outcomes [13,31], illustrating the important role of substrate and mineral interactions in taphonomy. Understanding these roles is crucial to determining and reducing biases, especially in settings of high diagenetic alteration and low fossil abundance (e.g. the Precambrian) [13]. As shown by the second example in §3.2 above (figure 5), equations (2.14) and (2.22) provide a way to quantify differences in depositional and preservation conditions that are directly comparable between settings, providing a unifying mathematical way to characterize biases in the fossil record.

As for the Price equation, equation (2.22) is not a mechanistic model and cannot directly predict the changes between the two entities; instead, it is an abstract, mathematically constrained expression of these changes [28]. Because of this universalistic nature (effectively simply describing the changes between entities A and B), the Price equation has been adapted for many fields outside evolutionary biology (e.g. [39–44]). The present expression (equation (2.22)), applied to the study of taphonomic change, offers a framework to describe and partition degradation both in natural and experimental conditions (when both the starting conditions and the derived state are known or can be estimated) with which measurements from any metrics can be incorporated. For example, work from Gibson *et al.* [17] on the experimental decay of sea anemones relied on artificially (although carefully selected) binned decay stages (four stages associated with 0–25%, 25–50%, 50–75% and 75–100% loss of the feature). Using the formalism presented here, these stages can be redefined as continuous variables. Each of their four characters of interest (anterior region, body tissue, outer dermal and gut) can be associated with a state value, for example, corresponding to the remaining percentage of the feature compared with the original state. This allows an illustration of the loss of feature over time, just as Gibson *et al.* have demonstrated, but with one major difference. Because it only relied on the frequency of objects and their state value, it is possible to apply it to widely morphologically diverging organisms under the same experimental conditions (for example, a shrimp and a sea anemone). For a selected metric, the present expression would then offer a description of their respective changes that are directly comparable, in addition to providing an insight into the respective decay dynamics of each organism.

In some situations, the loss of features during diagenesis follows a consistent sequence even across various environmental conditions, resulting in a morphological regression to the ancestral state [33,45,46]. This ‘stem-ward slippage’ has been observed in cephalochordate and cyclostomes [33,45], whereas other organisms may show no evidence for such patterns of degradation [13]. If morphological characters, labelled as plesiomorphic or apomorphic, were to be assigned a value according to a characteristic of interest, equation (2.14) or (2.22) could partition the effect of stem-ward slippage between the selective preservation of plesiomorphic characters (accounted by the first term of equations (2.14) and (2.22)) and the alteration of the character values during decay. More interestingly, perhaps, we could assign to each character a value corresponding to its degree of ancestry (e.g. the synapomorphic rank in [33]), providing an average plesiomorphic score for one organism. The evolution of this score can be tracked along decay in the experimental setting using equation (2.14) or (2.22), effectively recording the stem-ward slippage (§3.2; figure 6). If their degrees of ancestry are hierarchically comparable, the use of the present formalism permits direct comparison between organisms from different phylogenetic origins and can clarify some unclear taxonomic affinities, as with *Hardistiella* above [33].

The present formalism also constitutes an interesting new chemometric tool for molecular palaeobiology. Equation (2.22) is directly applicable to vibrational spectroscopy data, for example infrared spectroscopy. Infrared spectroscopy, notably Fourier transform (FTIR), identifies chemical bonds in a sample by detecting their vibrational modes, yielding information about their molecular composition and structure in the form of an absorption (or reflectance) spectrum. The technique has been applied for degradation experiments of organisms (e.g. [47,48]) or the thermal degradation of chemical and molecular compounds (e.g. [5,18]). By considering each band of interest in this spectrum as an ‘object’ and its measured parameters (e.g. peak intensity, width and position) as the possible state values, we can track the evolution of the signal partition (the part owing to the appearance and disappearance of absorption bands and the part owing to the changes in band parameter values) during degradation of a sample in a simple and consistent manner and compare this partition for each parameter. The evolution of the spectrum of a decaying organism may be very different for different organisms decaying under different conditions, but our formalism allows all the data to be unified in a common expression for the extent of taphonomic change.

The present work, by mathematically describing and partitioning the effect of post-mortem changes, constitutes a widely extendable approach for the study of taphonomical dynamics and the characterization of taphonomical systems (taphonometrics), with promising possible future applications in palaeoenvironmental, archaeological or forensic sciences.

## 5. Conclusion

Constraining the fossilization processes is a challenging task requiring the understanding of many changing multivariate parameters, most often specific to individual environmental settings and conditions. Here, we have shown that the passage from an organism to a fossil could be abstracted into a change between two collections of objects and partitioned into three terms, two of which constitute the terms of the classic equation of George Price. The last term accounts for the presence (migration from the sediment and *in situ* condensation) of new material within the fossil. Altogether, they provide a mathematical definition of fossilization and taphonomy and a framework that can be applied to describe any system at a molecular, cellular, organism or population scale. Since its first publication, the Price equation and its variations have been applied in many fields, from biology to statistical physics. It was yet to be extended to palaeontology and taphonomy.

## Data Availability

All data are available in the main text or the supplementary material [49].
